# Survey Study among Dentist in Bulgaria to Develop a Model for Risk Management in Dental Practice

**DOI:** 10.1155/2022/6142445

**Published:** 2022-06-06

**Authors:** Tihomira Schiller, Nina Musurlieva, Tanya Bozhkova, Veselina Kondeva, Mariana Dimitrova, Sevda Rimalovska

**Affiliations:** ^1^Department of Social Medicine and Public Health, Faculty of Public Health, Medical University-Plovdiv, Bulgaria; ^2^Department of Prosthetic Dentistry, Faculty of Dental Medicine, Medical University-Plovdiv, Bulgaria; ^3^Department of Pediatric Dentistry, Faculty of Public Health, Medical University-Plovdiv, Bulgaria

## Abstract

**Background:**

Quality control and risk management in the field of dental services is an important part of improving patient safety as well as that of the dentists. The introduction of a risk management model would simplify and facilitate this process.

**Aim:**

The aim of the study is to gather information about the structurе and organization of work processes in Bulgarian dental practices, which will serve as a basis for building a risk management model. *Material and Methods*. A survey was conducted among randomly selected dental associations in Bulgaria-Plovdiv, Sofia, Varna, and Burgas through an anonymized questionnaire, containing 30 questions. The respondents meet the main criteria, namely, to be dentists and to practice in Bulgaria. The study includes demographic data, information on the attitude of Bulgarian dentists towards issues related to quality management, and safety and risk in respect to dental practice. The results have been processed and analyzed through descriptive and graphical analysis using the statistical program SPSS 20.0.

**Results:**

A total of 103 Bulgarian dentists took part in the study, out of which 25.24% ± 4.28% have acquired a specialty. Women are 52.43% ± 4.92%, and men −47.57% ± 4.92%. The largest is the relative share of the respondents in the age range of 25-35 years −63.10% ± 4.75% and with work experience of 6-15 years −52.43% ± 4.92%. Most of the respondents do not define in writing the main tasks and activities −52.43% ± 4.92%, and do not use checklists in their practice -54.73%. The majority of the respondents do not hold regular meetings with their teams −50.49% ± 4.93%, as well as they do not conduct surveys among their patients −68.93% ± 4.56%. The majority of the respondents −41.75% ± 4.86% are guided by their personal judgment in respect to whether the written information provided to patients is comprehensible and accessible. The majority of dentists −45.63% ± 4.91% take informed consent only for expensive procedures in written and oral form −53.40% ± 4.92%. Out of all the respondents, 75.73% ± 4.22% have not analyzed the risk of slipping in their practices for the last two years.

**Conclusion:**

There is a lack of written definition of the main tasks and processes, as well as no use of checklists in the practices of most of the interviewed dentists. Meetings with teams are held irregularly. There is a lack of surveys among patients, as well as no objective feedback from patients regarding the comprehensibility of the information materials provided. Informed consent is obtained from patients mainly in written and oral form and only for costly manipulations. In the practices of most of the interviewed dentists, there has been no assessment of the risk of slipping and falling for the last 2 years.

## 1. Introduction

The quality and safety of the services offered in dental practices are extremely important for the success of the treatment and the satisfaction of patients. The meaning and application of this concept should be understood by each member of the medical team and introduced into the work process in the form of a risk management model. The implementation of such a model is of great importance not only for large medical and dental centers but also for smaller dental practices. At its core, risk and security management is expressed in the systematic, planned, and coordinated interaction of the individual components, as well as in the control, correction, and optimization of work processes [1]. This, in turn, leads to improved quality of dental services, increased patient safety, unproblematic and efficient work processes, better treatment results, and greater satisfaction for both patients and medical staff [2].

In Bulgaria, in State Gazette issue 41 of 08.05.2020, the general and the special rules for good medical practice of dentists were promulgated. In the form of recommendations and guidelines for all dentists practicing in the Republic of Bulgaria, they cover in general the rules for the professional conduct of dentists, the professional and ethical relationships with patients and the professional relationships with colleagues. Good dental practice maintenance is also featured, where dentists are obliged to regularly update their knowledge through postgraduate training, to maintain and improve the quality of their activities, as well as to know well the normative documents, related to dental practice [3]. The special rules are in the form of guidelines for work in the diagnosis and treatment of caries and its complications, of diseases of the mucous membranes and periodontium in adults and children, of endodontic, prosthetic, surgical, orthodontic treatment, etc. However, so far, there has been no developed structured model for risk management that meets the needs of dental practices in Bulgaria. The purpose of this study is to gather information about the needs and specifics of the activities of dentists in order to create a model for risk management in dental practices in Bulgaria.

## 2. Methods

The primary information needed for the purposes of the study was collected through a sociological method—a survey. An anonymized questionnaire in electronic version, developed for the needs of the research, was used. A pilot study was conducted in October 2021, including 20 dentists from four regional cities—Sofia, Plovdiv, Varna, and Burgas—in order to test and improve the methodology for collecting information, as well as to test the effectiveness of the selected information collection method. The minimum sample size of participants was determined based on power analysis for sample-size calculation.

The study was conducted in Bulgaria in the period November 2021-January 2022 with the personal participation and control of the researcher.

The used questionnaire consisted of 30 questions, which the respondents answered, after having previously been given instructions in relation to the survey and on how to fill in the questionnaire correctly. All questions are closed, and with question number 7-11, 14, and 18-24, the participants could choose more than one answer. The rest of the questions have only one possible answer.

The reliability of the questionnaire was established by assessing the internal consistency and by the test-retest method. In the correlation analysis, Cronbach's coefficient *α* is 0.856, and the Spearman-Brown coefficient rsb = 0.732 was calculated. Their high values confirm the reliability. The changing factors were studied using the Pearson (*r*) and Spearman-Brown (rsb) coefficients. An item analysis was made, calculating the difficulty and discriminatory power of the questions.

The data were entered and processed with the IBM SPSS Statistics 20.0 statistical package. Descriptive statistics for quantitatively measurable quantities and nonparametric (Pearson criterion) test were used to test hypotheses. For a significance level at which the null hypothesis was rejected, *p* < 0.05 was chosen.

## 3. Results

A total of 103 dentists practicing on the territory of the Republic of Bulgaria took part in the survey, out of whom 25.24% ± 4.28% have acquired a specialty. The relative share of the women participants was 52.43% ± 4.92%, and of the men −47.57% ± 4.92%. The largest is the relative share of the respondents in the age range of 25-35 years −63.10% ± 4.75% and with work experience of 6-15 years −52.43% ± 4.92%. The demographic characteristics of the contingent are presented in a summary form in Table 1.

When asked how responsibilities and obligations were distributed in their practices and whether they were defined in writing, only 2.91% ± 1.66% of respondents were established to have distributed the responsibilities in their practices entirely in writing, and 39.80% ± 4.82%—entirely orally. The majority of respondents −52.43% ± 4.92% answered that they do not define in writing the main tasks and activities. The results are presented in Table 2.

The processed data from the survey showed that the majority of respondents do not use checklists in their practice 54.73%. Those who have introduced checklists use them mostly for taking anamnesis (28.16% ± 4.43%) and for conducting hygienic measures (22.33% ± 4.10%). The results are presented in Table 3.

Out of the interviewed dentists, 27.18% ± 4.38% hold regular meetings with their teams, with the majority organizing such meetings irregularly 50.49% ± 4.93%. 22.33% ± 4.10% gave a negative answer. 78.64% ± 4.04% of the members of the dental teams take part in postgraduate trainings, and 21.36% ± 4.04% do not do it.

When asked whether surveys were conducted among patients, only 7.77% ± 2.64% of respondents answered that they use this method of feedback continuously or at regular intervals. The majority of the study participants 68.93% ± 4.56% gave a negative answer, and 71.84% ± 4.43% of the dentists noted that for the last 3 years they have not conducted any inquiries among patients. The responses are presented in a tabular manner in Table 4.

55.34% ± 4.90% of the dentists have established certain rules for action in case of patient complaints, and 44.66% ± 4.90% have not introduced such rules. When asked how they assess whether the written information provided is accessible and comprehensible to the patient, the majority of the respondents −41.75% ± 4.86%, said they were guided by their personal judgment, 24.27% ± 4.22% of the studied consulted the opinion of individual patients, and only 4.85% ± 2.18% used questionnaires for feedback; 23.30% ± 4.17% of respondents had not taken such measures up to then (Table 5).

The majority of the participants in the survey stated that they take informed consent only for expensive procedures (45.63% ± 4.91%) in written and oral form (53.40% ± 4.92%). The results are illustrated in Table 6.

The survey data show that 41.75% ± 4.86% of the respondents do not have rules for action at potential risk (e.g., polymorbid patients), and 35.92% ± 4.73 have not introduced any for coping pain (Table 7.)

To the question from the questionnaire, whether the risk of slipping and falling has been analyzed for the last two years, most of the dentists gave a negative answer −75.73% ± 4.22% (Table 8).

## 4. Discussion

The written definition of the responsibilities and tasks for the members of the medical team is an important part of the structure of the quality and risk management systems, as the tasks and responsibilities delegated in writing can always be controlled and changed and adapted if necessary. They are a way to achieve order and organization in everyday work, as each member of the team is aware of their responsibilities in writing. In the conducted survey, it is worth noting that the majority of the respondents −52.43% ± 4.92% answered that they do not define in writing the main tasks and activities. This result can be explained by the fact that a large part of the dentists in Bulgaria work independently in their practices, and there is no large turnover of staff. Routine staff know the processes and do not feel the need to document them. However, in the event of training new staff, the written definition of work processes would facilitate and shorten the start-of-employment period.

The need for and the benefits of checklists in medical and dental practice have been proven in a number of studies. In 2009, the National Patient Safety Agency in England published a study showing a significant reduction in the levels of postoperative complications or death from the use of surgical checklists [4]. This study clearly shows the benefits of using checklists and they are beginning to be used in more and more medical fields in order to increase patient safety. A study conducted by Ragusa et al. established that the introduction of surgical checklists reduces the risk of medical error in surgery [5]. In the field of dentistry, studies can be cited on the benefits of using checklists to reduce the risk of extraction of the wrong tooth [6] in endodontic or implant treatments [7, 8]. All these data point out the advantages of using checklists. In addition, checklists are an important mechanism for improving patient safety and reducing the risk of adverse events. The results of the study showed that most of the interviewed dentists do not use checklists 57.37%.

A significant part of building a successful risk management model is the effective communication in the medical team. Often, the consequences of impaired communication are, in fact, adverse events such as forgotten instruments and gauze in the surgical field [9] or surgical interventions on the wrong limb/organ or the wrong patient [10]. Methods for improving the communication between the members of medical teams are the regular meetings, where both—problems arising from work practice and ideas for optimizing the work process—can be discussed, responsibilities can be distributed, and new tasks can be delegated. In the current survey, the majority of respondents do not hold regular meetings with their team −50.49% ± 4.93%.

Patient feedback is essential to building a good risk management model. Patient satisfaction, recommendations, and patient complaints are valuable tools for improving safety. Studies from different countries have demonstrated the benefits of using the survey method to obtain objective feedback from patients on issues of treatment satisfaction [11]. The results of the present study established that only 7.77% ±2.64% conduct surveys among their patients continuously or at regular intervals as a method of feedback, and this is of paramount importance for improving the quality of dental services.

The comprehensibility and reliability of the information provided by the dentist are important aspects for the informed choice of the patient. The majority of the respondents rely on their personal judgment as a method of assessing the comprehensibility of the offered information −41.75% ± 4.86%. This could not be described as an objective method of assessment because the doctor, as a specialist in their field, and the patient, on the other hand, would have different perceptions and understanding of the information materials. 23.30% ± 4.17% of the respondents do not take such measures. Due to the lack of a method for assessing the comprehensibility of written information, the informed choice of the patient could not be guaranteed.

For dentists practicing on the territory of the Republic of Bulgaria, obtaining informed consent is legally regulated, and for almost all activities within the range of dental treatment, it must be in writing [12–15]. Lack of such can lead to financial sanctions or even to temporary deprivation of the right to practice the profession. The majority of the participants in the survey stated that they take informed consent only for expensive procedures (45.63% ± 4.91%) in a written and oral form (53.40% ± 4.92%).

The development of a risk management system in respect to dental practice requires also the working up of rules for dealing with medical emergencies, safety of the used and prescribed medication, and coping with pain [16]. The Bulgarian legislator has given the definition of emergency [17] and dental practices, as outpatient hospitals are obliged to provide the possible volume of medical activities to maintain the patient's vital functions until the arrival of an emergency medical team. It can be concluded that dentists must have in place rules of practice for at-risk patients, they must be able to recognize them and be prepared to cope with emergencies. In the current study, 41.75% ± 4.86% of respondents do not have rules for action at potential risk, and 35.92% ± 4.73 have not introduced rules for coping with pain.

Assessing the risk of falling should begin with identifying the patients most at risk in such cases. These are often older patients or people with diseases of the vestibular system, paralysis and paresis, and diseases of the visual system and others. Some medicines can also increase the risk of falls and injuries, e.g., medicines with psychopharmacological substances and opioid analgesics. The accessibility of the practices, the presence of large stairs, and the presence or absence of an elevator are just some of the aspects that are included in the risk analysis of falling in practices.

The results from the answers to this question show that the majority of respondents are not aware of the seriousness of the consequences from a possible fall and injury of the patient in a dental practice. Such consequences can be associated with serious physical conditions such as limb fractures, injuries, and head injuries requiring hospitalization, and more.

A limitation of this study is the small number of participants.

## 5. Conclusion

In conclusion, the conducted study showed that there is no written definition of the main tasks and processes in the practices of the majority of the interviewed dentists, as well as no use of checklists. More than half of the respondents do not meet regularly with their team. There is a lack of surveys among patients with most of the study-involved subjects, as well as no objective feedback from patients regarding the comprehensibility of the provided information materials. The majority of the respondents obtain informed consent from their patients in writing and oral but only in case of expensive manipulations. In the practices of most of the interviewed dentists, there has been no assessment of the risk of slipping and falling for the last 2 years.

## Figures and Tables

**Table 1:** Demographic data.

	Number (*n*)	Percentage (%)	Sp
Age			
25-35	65	63.10	4.75
36-45	22	21.36	4.04
46-65	14	13.59	3.38
>65	2	1.94	1.36
Gender	Number (*n*)	Percentage (%)	Sp
Female	54	52.43	4.92
Male	49	47.57	4.92
Work experience	Number (*n*)	Percentage (%)	Sp
<5	28	27.18	4.38
6-15	54	52.43	4.92
16-30	13	12.62	3.27
>30	8	7.77	2.64
Total	103	100.0	—

**Table 2:** Distribution of responsibilities and main tasks definition in a written form.

	Number (*n*)	Percentage (%)	Sp
Distribution of responsibilities			
Only in a written form	3	2.91	1.66
Written and oral	59	57.28	4.87
Only oral	41	39.80	4.82
Main tasks definition in a written form	Number (*n*)	Percentage (%)	Sp
Yes	49	47.57	4.92
No	54	52.43	4.92
Total	103	100.0	—

**Table 3:** Use of checklists in dental practices.

	Number (*n*)	Percentage (%)	Sp
Checklists use			
Anamnesis taking	29	28.16	4.43
Control of emergency equipment	20	19.42	3.90
Preparation of the necessary tools	20	19.42	3.90
Conducting hygienic measures	27	26.21	4.33
Other activities	23	22.33	4.10
Lack of introduced checklists	56	54.37	4.91

**Table 4:** Conducting surveys among patients.

	Number (*n*)	Percentage (%)	Sp
Conducting surveys among patients			
Yes, all the time	5	4.85	2.12
Yes, at regular intervals	3	2.92	1.66
Yes, but not regularly	24	23.30	4.17
No	71	68.93	4.56
Conducting surveys among patients over the last 3 years	Number (*n*)	Percentage (%)	Sp
Many times	12	11.65	3.16
Once only	17	16.50	3.66
Not conducted over the last 3 years	74	71.84	4.43
Total	103	100.0	—

**Table 5:** Check on the information provided as comprehensible to the patient or not.

	Number (*n*)	Percentage (%)	Sp
The written information provided is comprehensible to the patient			
At personal discretion	43	41.75	4.86
Inquiry of individual patients	25	24.27	4.22
Feedback from the questionnaires	5	4.85	2.18
External evaluation assistance	2	1.94	1.36
Other	4	3.88	1.90
No such measures have been taken so far	24	23.30	4.17

**Table 6:** Obtaining informed consent and form of the informed consent.

	Number (*n*)	Percentage (%)	Sp
Obtaining informed consent			
Yes, always for each procedure	44	42.72	4.87
Only for expensive procedures	47	45.63	4.91
No	12	11.65	3.16
Form of the informed consent.	Number (*n*)	Percentage (%)	Sp
Written	37	35.92	4.73
Oral	11	10.68	3.04
Written and oral	55	53.40	4.92
Total	103	100.0	—

**Table 7:** Rules for action at potential risk and when coping with pain.

	Number (*n*)	Percentage (%)	Sp
Rules of action in the presence of potential risk			
Yes	60	58.25	4.86
No	43	41.75	4.86
Rules for coping with patient pain	Number (*n*)	Percentage (%)	Sp
Yes	66	64.08	4.73
No	37	35.92	4.73
Total	103	100.0	—

**Table 8:** Analysis of the risk of slipping for the last two years.

	Number (*n*)	Percentage (%)	Sp
Analysis of the risk of slipping for the last two years			
Yes, in all rooms	13	12.62	3.27
Yes, in some rooms	12	11.65	3.16
No	78	75.73	4.22
Total	103	100.0	—

## Data Availability

The authors may provide any necessary information.
